# Defect Density and Atomic Defect Recognition in the Middle Layer of a Trilayer MoS_2_ Stack

**DOI:** 10.1021/acs.nanolett.4c02391

**Published:** 2024-07-01

**Authors:** Moritz Quincke, Manuel Mundszinger, Johannes Biskupek, Ute Kaiser

**Affiliations:** † Central Facility Materials Science Electron Microscopy, Ulm University, 89081 Ulm, Germany; ‡ Institute for Quantum Optics, Ulm University, 89081 Ulm, Germany

**Keywords:** Few-layer MoS_2_, Sulfur vacancy, Defect recognition, damage cross-section, low-voltage C_C_/C_S_-corrected HRTEM, FIB cross-section

## Abstract

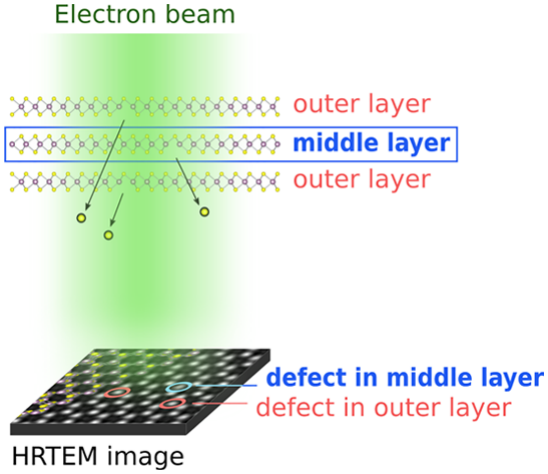

Molybdenum disulfide (MoS_2_) is one of the most intriguing two-dimensional materials, and moreover, its single atomic defects can significantly alter the properties. These defects can be both imaged and engineered using spherical and chromatic aberration-corrected high-resolution transmission electron microscopy (C_C_/C_S_-corrected HRTEM). In a few-layer stack, several atoms are vertically aligned in one atomic column. Therefore, it is challenging to determine the positions of missing atoms and the damage cross-section, particularly in the not directly accessible middle layers. In this study, we introduce a technique for extracting subtle intensity differences in C_C_/C_S_-corrected HRTEM images. By exploiting the crystal structure of the material, our method discerns chalcogen vacancies even in the middle layer of trilayer MoS_2_. We found that in trilayer MoS_2_ the middle layer’s damage cross-section is about ten times lower than that in the monolayer. Our findings could be essential for the application of few-layer MoS_2_ in nanodevices.

The scientific community has shown increasing interest in the unique optical and electronic properties of atomically thin two-dimensional (2D) materials.^1−4^ These materials span a wide spectrum, ranging from insulating over semiconducting to metallic characteristics, and exhibit quantum phenomena such as superconductivity or charge density waves.^5,6^ Among the 2D materials, few-layer transition metal dichalcogenides (TMDs) are promising candidates for technological applications in nanodevices such as memristors,^7^ field effect transistors^8^ and tunneling devices.^9−13^ The properties of TMDs are highly dependent on the number of layers, as for example, some TMDs undergo a transition from indirect to direct bandgap when reduced from few-layer to monolayer configurations.^14^ Additionally, tuning the properties of TMDs can be accomplished by engineering of atomic defects.^15−18^ While the atomic structure and mechanisms of defect creation in monolayer materials have been extensively studied,^19−24^ the knowledge of defects in few-layer materials is crucial as well.^25−27^ For example, defects in few-layer TMDs can be used as single photon sources.^28,29^ Moreover, in tunneling devices, the vertical position of defects defines both the capacitance to the source and drain and the respective tunneling rates.^10^ In the case of a superconductor, the vertical position of defects defines the proximity between the defect and the superconductor.^11^ However, the precise defect structure of few-layer TMDs, in particular in its vertical direction, remains unknown.

Aberration-corrected (scanning) transmission electron microscopy ((S)TEM) enables atomic resolution imaging of 2D materials, allowing clear identification of atomic defects in monolayers.^30,31^ However, for few-layer materials, interpreting (S)TEM images becomes more difficult. These images represent a two-dimensional projection of a three-dimensional object, displaying the integrated contrast of multiple atoms stacked within one atomic column. A single missing atom in a column containing many atoms alters the contrast only slightly, which complicates the differentiation between intact columns and defective columns. Additionally, pinpointing defects in specific layers within a few-layer flake is challenging. To examine these layers, cross-sectional lamellae can be prepared by focused ion beam (FIB) milling.^32^ However, imaging single atomic defects in cross-sectional HRTEM images of few-layer samples is impossible due to random orientation of the material on the substrate and its relatively high thickness. With (S)TEM, atomic defects can not only be imaged, but also created due to the impact of electron beam irradiation.^22^ In TMDs, minimizing uncontrolled electron-beam-induced damage can be achieved by using electron energies below 80 keV. Such low energies, achieved using the spherical and chromatic aberration-corrected (C_C_/C_S_-corrected) *SALVE* instrument,^33,34^ result in a fine balance between imaging the pristine structure or dose-controlled defect engineering. In high-resolution (HR)TEM, the whole field of view is illuminated with a broad, parallel electron beam, enabling to capture large field-of-view images containing thousands of atoms, thus providing robust statistical data. Moreover, the brief frame acquisition time of about one second in TEM allows capturing of fast image series that represent the dynamics of defect creation. This capability is crucial for studying the real-time evolution of material properties under electron irradiation.

In MoS_2_, electron-beam-induced damage differently affects Mo and S atoms. Given the 560 keV knock-on damage threshold for Mo atoms, displacement during imaging with 80 keV electrons is highly unlikely.^42^ The knock-on damage threshold for the much lighter S atoms is about 90 keV.^42^ S atoms can be removed even at voltages below this threshold due to lattice vibrations and electronic excitations.^12^ At 80 keV electron energy, two different defects are created in MoS_2_: (1) the single S-vacancy, where one S atom and (2) the double S-vacancy, where two S atoms in one column are sputtered away. These defects alter the electronic and physical properties of MoS_2_, which can significantly impact its function in various applications, particularly in electronics and catalysis.

HRTEM image series can provide valuable insight into how fast the electron-beam-induced damage takes place. This can be quantified by the damage cross-section, defined as σ=ΔV/(N*ϕ), where Δ*V* is the number of S atoms removed by a certain electron dose ϕ, and N is the total number of S atoms in the area being investigated. The damage cross-section is an important quantity in material science as it limits the stability of a material in the electron microscope and it allows for quantification of the defect density, based solely on the electron dose. In the past, damage cross sections were quantified by manual counting of defects in Fourier-filtered HRTEM images,^19^ which however is time-consuming and user-biased. Common state of the art approaches for automatic image evaluation of atomically resolved HRTEM/STEM images are deep learning^35−37^ and fitting with multiple Gaussians.^38−40^ However, for the existing approaches, defect recognition only works for monolayer TMDs and not for defect recognition in few-layer TMDs.

Here we introduce a method to discern the subtle intensity differences caused by defects in 1–3-layer MoS_2_, without relying on deep learning techniques. In contrast to deep learning approaches, the defect identification criteria are transparent and straightforward to understand. We utilize the precise contrasts in C_C_/C_S_-corrected HRTEM to capture the maximal intensities at the positions of the atomic columns. These intensities are refined by a local normalization procedure, allowing for a clear differentiation between the intensities of defective and intact atomic columns. Additionally, the stacking order of the crystal structure is determined by cross-sectional TEM and with this, defects are assigned to a certain layer, even to the middle layer in a trilayer MoS_2_ stack.

*Automatic Defect Recognition Algorithm*. The crystal structure of monolayer 2H MoS_2_, as seen from the top, is a hexagonal lattice where lattice sites with one Mo atom neighbor atomic columns with two S atoms stacked above each other. For clearer writing, in the following, lattice sites of single Mo atoms are also called “atomic columns”. More layers are added in a way that a Mo atom in the second layer is below the S atoms in the first layer. In Figure 1, the crystal structure of mono-, bi- and trilayer MoS_2_ is shown in both, plan and side views. In a plan-view HRTEM image of MoS_2_, the integrated intensity along the atomic columns is recorded. In the following text we refer to C_C_/C_S_-corrected HRTEM images simply as “HRTEM images”, as all images of this work were recorded with the C_C_/C_S_-corrected *SALVE* instrument.^33,34^ The crystal structure of 1–3-layer MoS_2_, in plan-view, can be divided into two substructures, as indicated by green “+” and blue “×” in the plan-view in Figure 1. The different filling characteristics of the atomic columns belonging to the two substructures can be seen when comparing the blue and green rectangles. In a monolayer, one substructure contains only S atoms and the other substructure contains only Mo atoms. In a bilayer one substructure contains atomic columns with S atoms only in the top layer and Mo atoms only in the bottom layer. In the other substructure it is the other way around and S atoms are only present in the bottom layer. Finally, in a trilayer one substructure contains S atoms only in the outer layers and the other substructure contains S atoms only in the middle layer.

**Figure 1:**
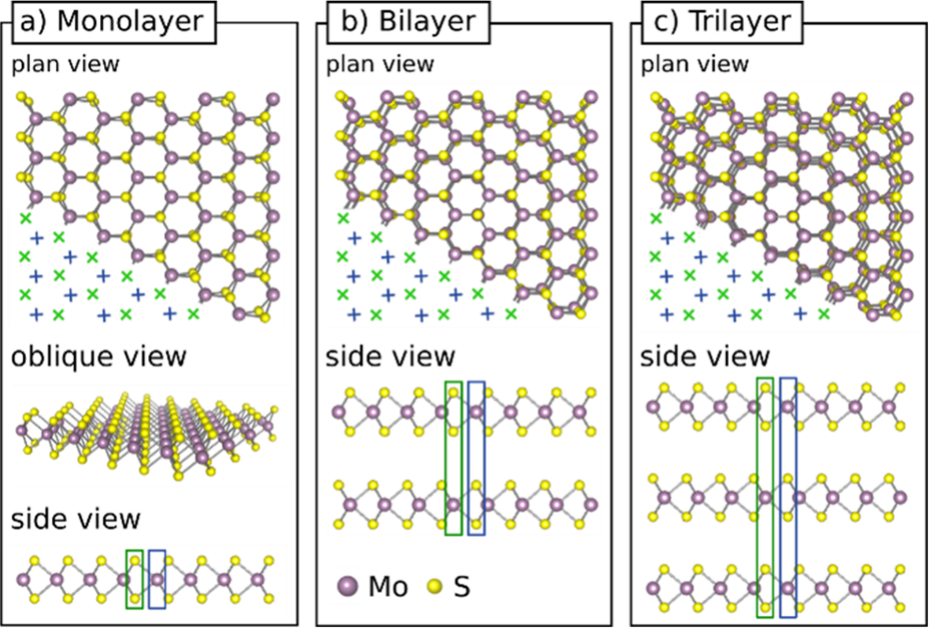
Crystal structure of pristine mono- and bi- and trilayer MoS_2_ from different perspectives. (a) For a monolayer, one can see that the overall hexagonal lattice can be divided into a substructure (S-substructure) only containing S atoms (blue box) and a substructure (Mo-substructure) only containing Mo atoms (green box). The two substructures are also indicated by the respective crosses in the plan view. (b) A bilayer on the other hand can be divided into a substructure containing S atoms in the top layer (blue box) and another substructure containing S atoms in the bottom layer (green box). (c) Finally, a trilayer can be divided into a substructure containing S atoms in the outer layers (blue box) and another substructure containing S atoms in the middle layer (green box). (Crystal structures designed with VESTA^43^).

The workflow to automatically detect S vacancies is shown exemplarily for a monolayer in Figure 2. In a first step, all individual atomic column positions, including those with very low intensities due to single or double S vacancies are obtained from the HRTEM image. Furthermore, the obtained positions are divided into the two substructures. The result is illustrated in Figure 2b. Next, the intensities at the previously obtained atomic column positions are extracted from a HRTEM image, treated with a Gaussian filter to remove contribution from Poisson noise of the electrons and readout noise of the electron detector. From the extracted intensities, an atomic column intensity histogram like in Figure 2c is created. In this histogram, the statistics of thousands of atomic columns can be exploited. In a plan-view HRTEM image of monolayer MoS_2_, the intensity of two S atoms stacked in one atomic column is similar to the intensity of one Mo atom. Therefore, both substructures cannot be differentiated in a HRTEM image of intact MoS_2_. By flipping the sample, both substructures would be exchanged and therefore the crystal structure’s orientation relative to the electron beam is unknown. However, the distribution in the histogram can differentiate between the two substructures because of the defect statistics. As explained above, the Mo atoms are stable under our experimental conditions and only S atoms are knocked out. For the case of a monolayer, the S-substructure can be identified by the counts for single S-vacancies, and double S-vacancies with intensities lower than the intact atomic columns. The respective intensity ranges are indicated by the double arrows in Figure 2c. The intensity ranges are automatically calculated from a Gaussian fit of the histogram peaks, shown in Figure S4. Atomic columns with intensities corresponding to single or double vacancies are identified and marked as defects in the HRTEM image, as shown in Figure 2d. In the histogram of the Mo-substructure there are no counts for intensities below the main peak of intact atomic columns, confirming that the Mo-substructure is perfectly intact. The defect recognition can also be applied for bi- or trilayer MoS_2_, where more atoms are stacked above each other in atomic columns. In this case, the relative change in intensity due to one missing atom is much smaller and a local normalization as discussed in Figure S8 becomes necessary.

**Figure 2:**
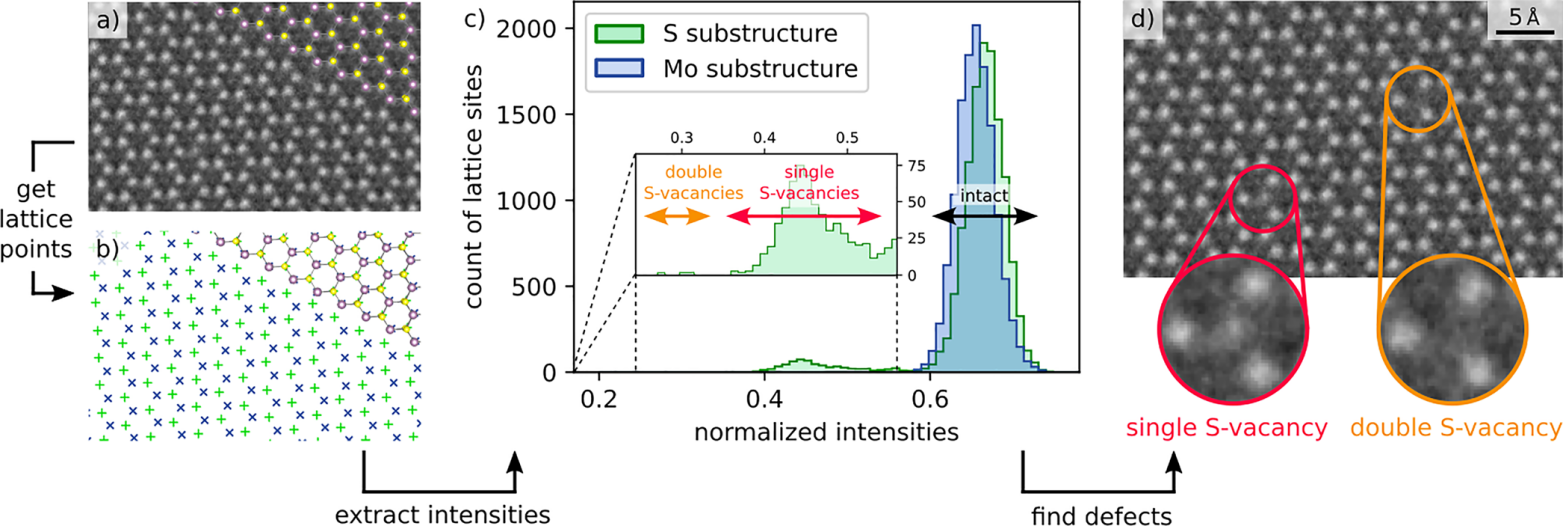
Defect recognition with the atomic column intensity histogram method exemplified for a monolayer as the simplest case: (a) Cutout of a C_C_/C_S_-corrected HRTEM image, partly overlaid with an atomic structure model. (b) Identification of atomic column positions and division into the two substructures introduced in Figure 1 (green “+” and blue “×”, details are given in Figures S2–4). (c) The intensity (gray-value) at each of the positions in (b) is extracted and plotted in an atomic column intensity histogram separately for the S-substructure (green “+” in (b)) and the Mo-substructure (blue “×” in (b)). There is one main peak of high intensities belonging to intact atomic columns for the Mo- and the S-substructure. For the S-substructure, there is a second peak corresponding to the intensity of single S-vacancies and additionally there are some counts for even lower intensities corresponding to double vacancies. As defects can also be created during the acquisition of one image, some counts at intermediate intensities are observed. (d) Image (from (a)) with marked single and double S-vacancies identified by the atomic column intensity histogram.

*Vertical Position of Defects in Bilayer and Trilayer MoS_2_*. The substructures in monolayer MoS_2_ can be assigned based on the defect distribution, as only S atoms are affected by beam damage and as only one substructure contains S atoms. In bi- and trilayer MoS_2_ both substructures contain S atoms (cf. Figure 1) and therefore the assignment of the two different substructures cannot be done only based on the defect distribution. The plan-view HRTEM images of 1–3-layer MoS_2_ used for the defect analysis in this work are all recorded from the same monocrystalline flake containing mono-, bi- and trilayer regions. However, the known substructures in the monolayer area cannot be related to the bi- and trilayer regions, as it is uncertain whether the second and third layer starts above or below the mono- or bilayer. To resolve this, a cross-sectional HRTEM image of the same flake was acquired. After recording the plan-view images from freestanding areas on a Quantifoil holey carbon grid, the sample was placed on a silicon substrate, and a vertical FIB lamella was cut out (see Figure S5). In Figure 3, plan-view and cross-sectional view HRTEM images of the same 1–3-layer MoS_2_ flake can be seen. The high magnification cross-sectional view images in Figure 3f and 3g clearly show that the new layer begins at the bottom for the transition from mono- to bilayer as well as for the transition from bi- to trilayer. Knowing this, the substructures in the bi- and trilayer images can be assigned unambiguously. The substructure which is the S substructure in the monolayer region, corresponds to the substructure containing S atoms in the top layer in the bilayer region. This configuration is illustrated in Figure 3h. In the same way the substructure containing S atoms in the top layer in the bilayer region, corresponds to the substructure containing S atoms in the outer layers in the trilayer region. With this information, the defects, identified from the intensity histogram method, can be assigned to the respective substructure, revealing information on their vertical position.

**Figure 3:**
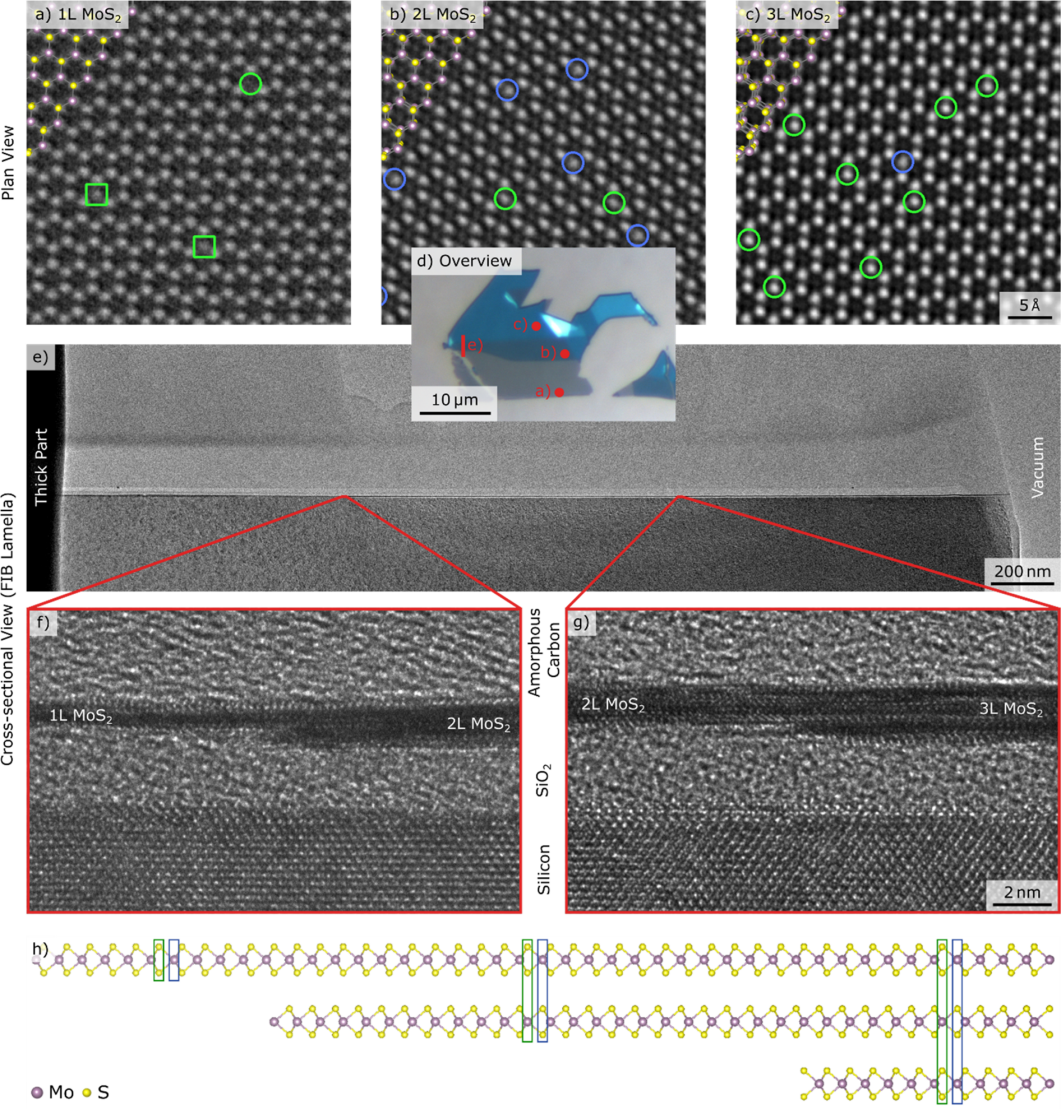
80 kV C_C_/C_S_-corrected HRTEM plan-view and cross-sectional view images of the same MoS_2_ flake containing the mono-, bi- and trilayer regions. (a-c) HRTEM images of mono-, bi- and trilayer MoS_2_. The green circle and squares mark sulfur double and single vacancies in the case of a monolayer. The blue and green circles mark, sulfur vacancies in the bottom and the top layer in the case of a bilayer and sulfur vacancies in the outer layers and middle layer in the case of a trilayer, respectively. (d) The used MoS_2_ flake as exfoliated on 90 nm SiO_2_. The red line marks the position of the FIB lamella and the red points mark the regions investigated in plan-view. (e) Overview bright-field TEM image of the FIB lamella. (f, g) Cross-sectional HRTEM images of the transition from mono- to bilayer and from bi- to trilayer MoS_2_ showing the direction of the step. (h) Crystal structure of 1–3-layer MoS_2_ in side view with the substructures marked in the respective colors. Details are given in Figures S5–7.

An atomic column intensity histogram of trilayer MoS_2_ can be seen in Figure 4a. The (blue) histogram of the substructure with S atoms in the middle layer shows some defects, but much fewer than the (green) substructure with S atoms in the outer layers. This proves that although the outer layers protect the atoms in the middle layer from being knocked out, defects are created in the middle layer. Single atomic defects in trilayer MoS_2_ manifest with a much weaker intensity difference, making them hardly visible to the naked eye in the HRTEM images. Therefore, in Figure 4b-e, alongside HRTEM images of defects, line scans are presented for comparison with HRTEM image simulations (find the simulated images in Figure S9). The experimental line fits are in good agreement with the simulations, demonstrating the effectiveness of the defect recognition algorithm presented here for up to three layers.

**Figure 4:**
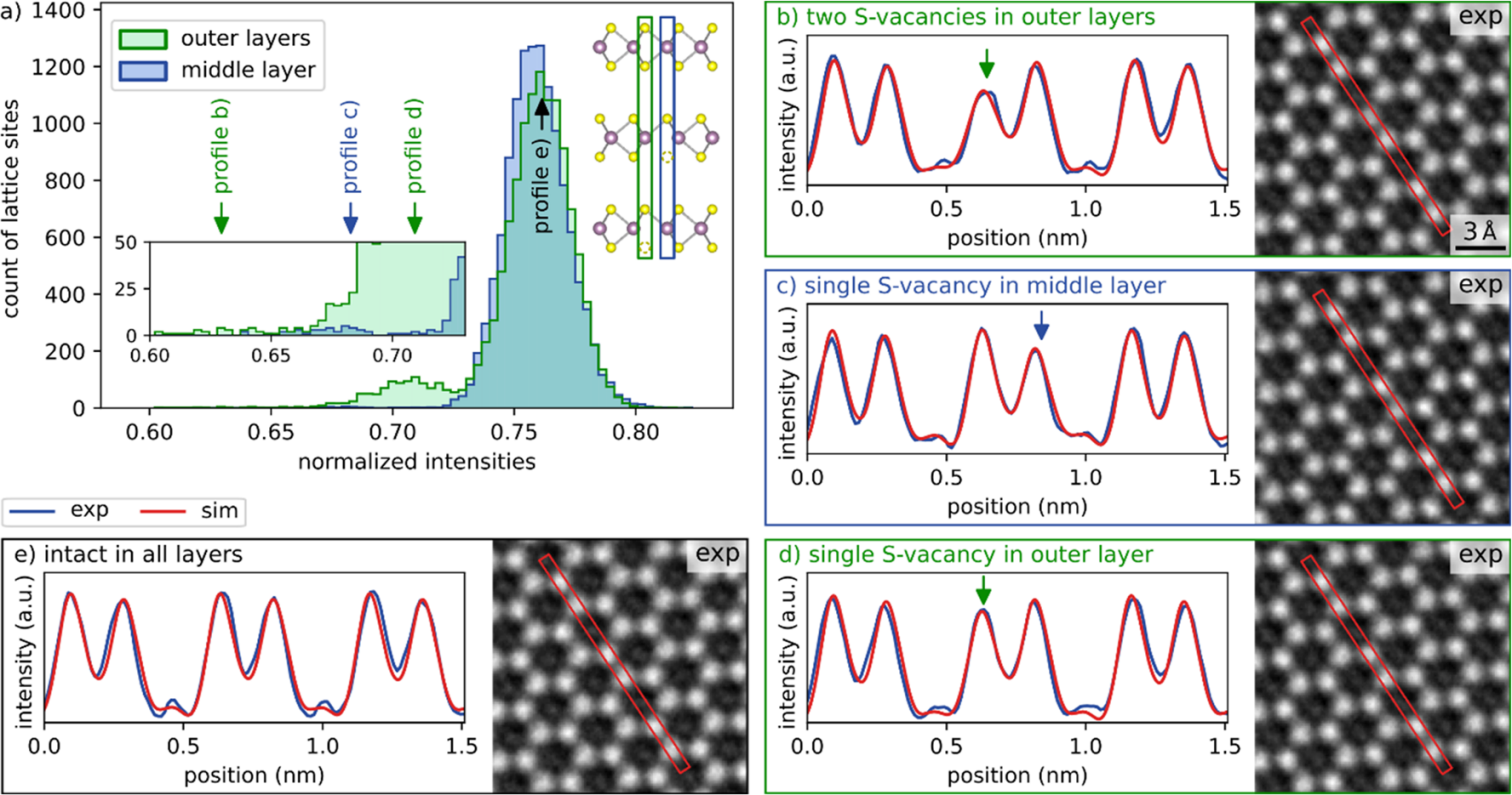
Comparison of experiment and simulation of different S vacancies in trilayer MoS_2_. (a) Atomic column intensity histogram of trilayer MoS_2_. The inset shows the crystal structure model in side view with a defect in the middle layer (green substructure) marked with a green box and a defect in an outer layer (blue substructure) marked with a blue box. (b-e) Exemplary line profiles with the corresponding intensities marked in (a). The experimental (exp) profile is extracted from the images on the right, respectively. HRTEM image simulations (sim) are plotted for proving the nature of the defects. The defoci for the simulation, matching the experiment are between −6.3 nm and −7.0 nm. The aberrations obtained from the experimental PCTF, used for the simulation are C_3_ = −7 μm, A_1_ = 1.05 nm, A_2_ = 15 nm and B_2_ = 10 nm. See also simulated images in Figure S9. The presence of triple and quadruple S vacancies is ruled out in Figure S10. The influence of reasonable variations of aberrations is small, still showing clear intensity differences for defects as evaluated in Figure S11. Further experimental support on the recognition of defects in trilayer is given in Figure S12. (The abtem software package was used for creating the simulated images.^45^)

*Vertical Position Dependent Damage Cross-Section*. To experimentally determine the damage cross-section, plan-view HRTEM image series of 1–3-layer MoS_2_ are recorded (See Figure S11). The images are recorded under continuous electron-beam exposure and as the accumulated dose increases from frame to frame, more and more defects are created. The starting dose (∼2 × 10^7^ e^–^nm^–2^s^–1^) is limited by the time needed for selecting a clean sample area and for focusing. The evaluation of the series is stopped as soon as the high defect density leads to defect structures which are more complex than isolated single or double sulfur vacancies. Exemplary atomic column intensity histograms of both substructures in 1–3-layer MoS_2_ are shown in the first two lines in Figure 5. They are always given at the beginning of the image series (filled plot) and for an exemplary higher accumulated dose (bold line).

**Figure 5:**
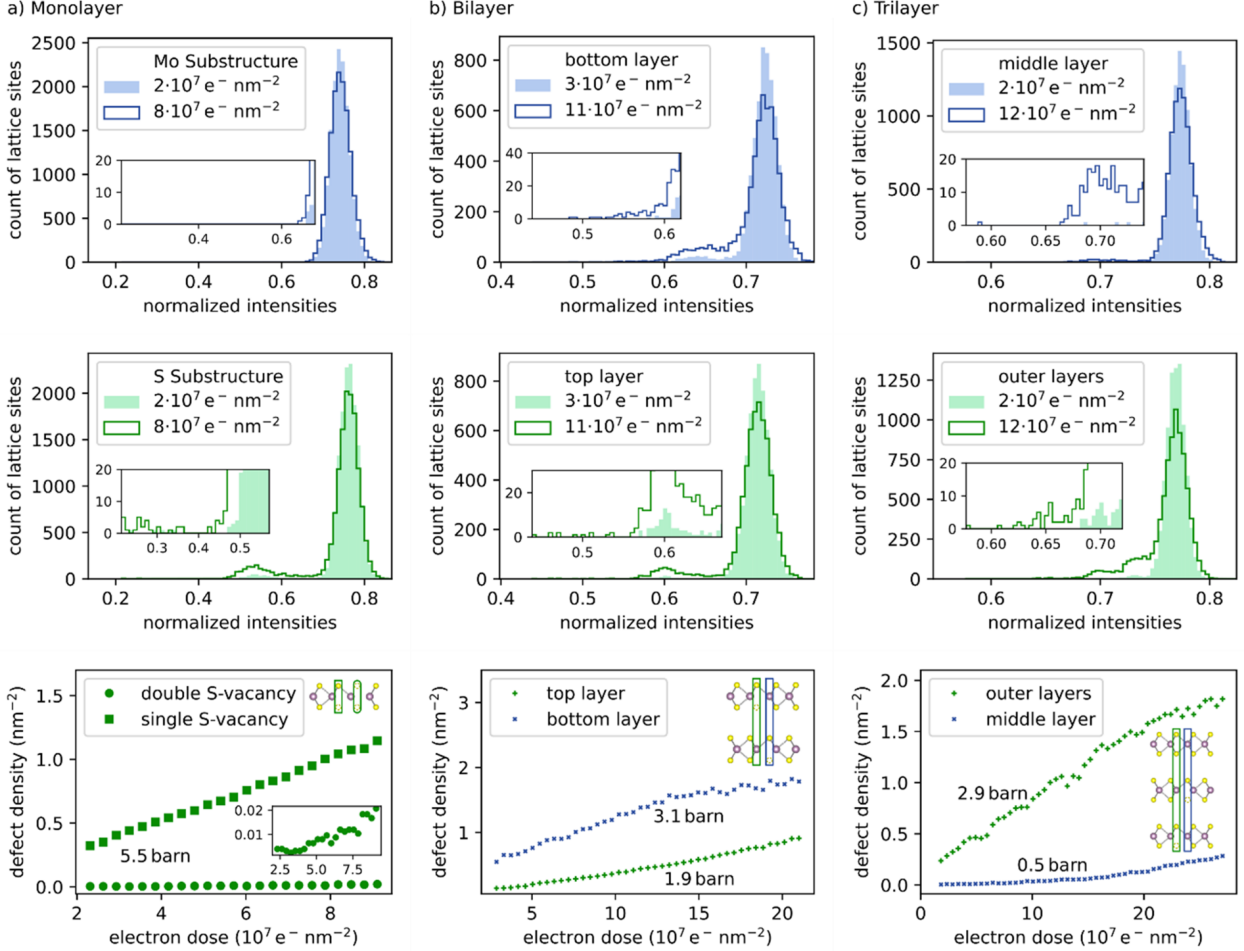
Vertical position-dependent defect dynamics in mono-, bi- and trilayer MoS_2_. (a) Monolayer: The histogram of the Mo substructure shows no defects from the start to the end frame. The histogram of the S substructure shows increasing single and double vacancies with increasing accumulated doses. The trend of defect density in the lower panel, is extracted from a time series of images. (b) Atomic column intensity histograms and trend of defect density for bilayer and (c) trilayer MoS_2_. The given damage cross sections are calculated from the average gradient of the defect density, counting double vacancies twice in the monolayer case. The exposure before acquiring the first frame is due to navigation to the target area and focusing and was tracked by a video, as explained in the supplementary methods section. Each series is stopping at doses before extended defects occur. The insets in the bottom row show a side-view crystal structure model with the two substructures marked in the respective colors.

From the atomic column intensity histograms in Figure 5, the separation of the peak containing intact atomic columns from the peak containing defective atomic columns can be extracted. The comparison of the histograms from different numbers of layers shows that this peak separation decreases with increasing numbers of layers. This is expected as the influence on the intensity from one missing atom is smaller when the atomic column contains more atoms. The discussed peak separation, evaluated from the defect statistics in a single plan-view HRTEM image, allows to identify the number of layers in the sample. As explained in,^41^ without exploiting the defect statistics only monolayer MoS_2_ can be identified.

In the intensity histograms in Figure 5, the area under the lower intensity peak corresponds to the number of defects and the area under the higher intensity peak corresponds to the number of intact atomic columns. Comparing the histograms at different accumulated doses (filled plot and bolt line), one can see qualitatively that with increasing electron dose the number of defects increases for all sulfur containing substructures in 1–3-layer. Correspondingly, the number of intact atomic columns decreases.

To quantitatively assess the damage cross-section, the defect density in each frame of the HRTEM image series is evaluated using the automatic defect recognition algorithm presented here. The defect densities dependent on the accumulated dose are plotted in the bottom line of Figure 5. From the slope in this plot the damage cross-section can be calculated. Therefore, our defect recognition algorithm can efficiently be used to automatically analyze HRTEM image series and obtain an experimental value for the damage cross-section. For the monolayer, which contains defects only in one substructure, single and double S vacancies are plotted separately. The obtained damage cross-section of 5.5 barn is in agreement with earlier results.^19^ Only about 1% of the detected defects are double vacancies, which shows that double vacancies do not significantly impact on the evaluation of the damage cross-section. Therefore, for bi- and trilayer MoS_2_, single and double vacancies will no longer be distinguished.

The structure of bilayer MoS_2_ makes it an ideal platform to study how defect creation depends on the orientation of the sample relative to the incoming electron beam. The damage cross-section for the layer averted from the electron beam (bottom layer) is higher than for the layer facing the electron beam (top layer). As the sample is transparent to the electron beam, the top layer cannot shield the incoming electrons from the bottom layer. However, in the beam averted layer, atoms can be sputtered in the same direction as the incoming electrons without any layer below, protecting against sputtering. At higher doses, the damage cross-section slightly increases for the top layer and decreases for the bottom layer. One possible explanation is that at higher defect densities, S atoms sputtered in the top layer in the direction of the electron beam can fill vacancies in the bottom layer.

On the other hand, trilayer MoS_2_ provides a perfect platform to study defect mechanisms in the middle layer. This layer is of special interest because it is shielded from beam damage by the outer layers. In a trilayer configuration, it is possible to distinguish between defects in the middle layer from those in the outer layers. However, it is not possible to distinguish between defects in the top layer and the bottom layer. The difference in defect creation between the top and bottom layers was already studied in the bilayer case. Figure 5c shows that defects are continuously created in the middle layer of trilayer MoS_2_ by electron beam irradiation, even though the middle layer is protected by the outer layers. The damage cross-section in the middle layer slightly increases with the accumulated dose, likely because atoms sputtered in the middle layer can fill more vacancies in the outer layers. The average damage cross-section over the observed period of time can be given as 0.5 barn. In literature, a protective effect on MoS_2_ was already experimentally shown with additional graphene layers^44^ but these results cannot be applied to the question how the outer MoS_2_ layers protect the middle layer. To the best of our knowledge, it was not previously shown whether beam-induced damage can occur in the middle layers of few-layer TMDs. The results were confirmed through additional HRTEM image series on the same sample and on another sample (Figures S14–16).

In summary, equipped with the knowledge of the crystal structure symmetry and utilizing our method for analyzing intensity statistics from C_C_/C_S_-corrected HRTEM images, we successfully identified the vertical locations of defects in mono, bi- and trilayer MoS_2_. Notably, this approach enabled also the identification of the nature of the defects and their density in the middle layer of a trilayer stack, where we observed a moderate level of electron-beam induced single and double sulfur vacancies. Our approach paves the way to precise engineering of specific defects and defect densities in designated layers of few-layer TMDs, which is crucial for determining their functionality and thus their usage for integration into various device applications.

## Data Availability

The Python code for the atomic defect recognition and exemplary raw data is available at 10.5281/zenodo.11065629.

## Supporting Information Available

Additional experimental details, materials, and methods, including image simulations and reproductions of the results (PDF)

## Abbreviations

2D: two-dimensional
MoS_2_: molybdenum disulfide
TEM: transmission electron microscopy
STEM: scanning TEM
HRTEM: high-resolution TEM
C_S_: spherical aberration
C_C_: chromatic aberration
TMD: transition metal dichalcogenide
FIB: focused ion beam
SALVE: subangstrom low-voltage electron microscopy
exp: experiment
sim: simulation
