# Supplementary material for: Defect Density and Atomic Defect Recognition in the Middle Layer of a Trilayer MoS2 Stack
Source: Nano Lett. 2024 Jul 1;24(34):10496–503. doi: 10.1021/acs.nanolett.4c02391 (PMC11363126; doi:10.1021/acs.nanolett.4c02391)
Supplement: Supplementary file 1 — nl4c02391_si_001.pdf [file nl4c02391_si_001.pdf]

Supplementary information to:

Defect density and atomic defect recognition in the middle layer of a trilayer MoS_2 stack

Moritz Quincke[1,2], Manuel Mundszinger[1], Johannes Biskupek[1], Ute Kaiser[1,2]

[1] *Central Facility Materials Science Electron Microscopy, Ulm University, 89081 Ulm, Germany*
[2] *Institute for Quantum Optics, Ulm University, 89081 Ulm, Germany*

Methods

TEM sample preparation. We purchased bulk crystals of MoS_2 from HQ Graphene (Groningen, Netherlands) and exfoliated them on a 90nm SiO_2/Si substrate using the scotch tape method. 1-3-layer regions were identified by optical contrast in an optical microscope.[1, 2] A standard QUANTIFOIL® gold grid with a R1.2/1.3 holey carbon film was placed on the MoS_2 flake. The flake is positioned and held in position by a glass needle mounted on a xyz-stage. A small droplet of isopropyl alcohol (IPA) was added to the grid. The IPA flows between the grid and the substrate and pulls the grid toward the substrate as it evaporates. The silicon was etched with a solution of potassium hydroxide (KOH) to release the flake from the substrate.[3] The flake was annealed at 200 °C for 15 min at ambient conditions just before loading it into the TEM.

Electron irradiation and plan view HRTEM image acquisition. The sub-Angström low-voltage electron microscope (SALVE) with image-side correction of chromatic and spherical aberration and an achievable information limit of 76 pm at an electron energy of 80 keV[4] was used to acquire the images. The values of C_S and C_C were tuned to around -10 µm, respectively. The images were acquired at the 4k x 4k FEI Ceta2 CMOS camera, sampled with 11.9 pm/px. Dose rates of 2-3 x 10^6 $e^-nm^{-2}s^{-1}$ were used to balance good signal to noise ratio and low beam damage. The applied electron dose rate is directly accessible from the integration time of a frame, the counts on the camara and the conversion rate of 15 counts per electron. For the calibration of the conversion rate, we estimate an error of approximately 5%. The dose rate was found to be constant within each image series. The image series were automatically recorded with an acquisition time of 0.25s and a frame rate of 3.1 Hz. The high frame rate allows to start the acquisition already during approaching the target area and focusing and therefore the total dose on the investigated area can be tracked. For the frames acquired after approaching and focusing (the actual image series) an alignment procedure, as illustrated in Figure S1, was used to compensate for slight sample drifts. To improve the signal to noise ratio, 4-8 frames were added to one image. As a post-specimen shutter was used during readout, the accumulated dose at a certain frame can be calculated from the dose rate and the totally elapsed time. In monolayer MoS_2 at higher accumulated electron doses (~$10^8e^-nm^{-2}$), the high defect density leads to the formation of S-vacancy-lines with shifted atom positions[5]. In this study the experiments are stopped before mirror-twin-boundaries are produced. The vacuum in the TEM column was below 10^{-5} Pa.

Local intensity normalization. As discussed in the main text, the intensity difference, arising from one missing S atom can be very faint. This is critical when analyzing images with a large field of view. Even a tiny tilt or bending of the material leads to varying imaging conditions over the whole field of view, resulting in a variation of the extracted intensities. Calculating the "atomic column intensity histogram" over the whole field of view, these shifts would lead to a broadening of the peaks, limiting the separation of the peak containing defective atomic columns from the peak containing intact atomic columns. As the evaluation of a preferably large field of view is necessary to get a good statistic for defect recognition, the variation of the extracted intensities over the field of view has to be corrected. A first rough normalization is achieved by removing the intensity gradient in the raw HRTEM image with simple image filtering methods. Finer normalization is achieved by normalizing the extracted intensity at each individual atomic column position by the local mean intensity at the surrounding atomic column positions in the same substructure. The local intensity normalization is fundamental to mitigate the broadening effect due to variations in imaging conditions across the field of view and is applied before creating all histograms shown in this work.

FIB lamella and cross-sectional view HRTEM image acquisition. The lamella was prepared in a Zeiss NVision 40 Ar (Zeiss Microscopy) FIB/SEM (focused ion beam/scanning electron microscope) system. The selected sample

region was in a first step carefully covered with a protection layer of carbon via the gas injection system (GIS) in the electron deposition mode at 5 kV acceleration voltage to ensure an unscathed sample. FIB-GIS deposition was then used to deposit the bulk carbon layer. The lamella preparation followed the standard recipe by cutting out the region of interest with the ion beam at high currents at 30 kV acceleration voltage, lifting out the sample volume via micro manipulator and attaching it via GIS deposition to a lift out grid. The thinning was carried out with gradually decreasing currents, for polishing of the lamella the 5 kV FIB mode with low current was used. The lamella on the lift out grid was inserted into the SALVE instrument and cross-sectional view HRTEM images were acquired. For details see Figures S5-7.

Design of crystal structures and HRTEM image simulation. We used VESTA[6] to create crystal structure files for the simulation and for the illustrations the figures. To simulate trilayer MoS_2 plan view HRTEM images, we applied abTEM, an open-source Python API based on the multislice algorithm.[7] We applied the parameters that we obtained experimentally before image acquisition.

Supplementary Information to Figure 2

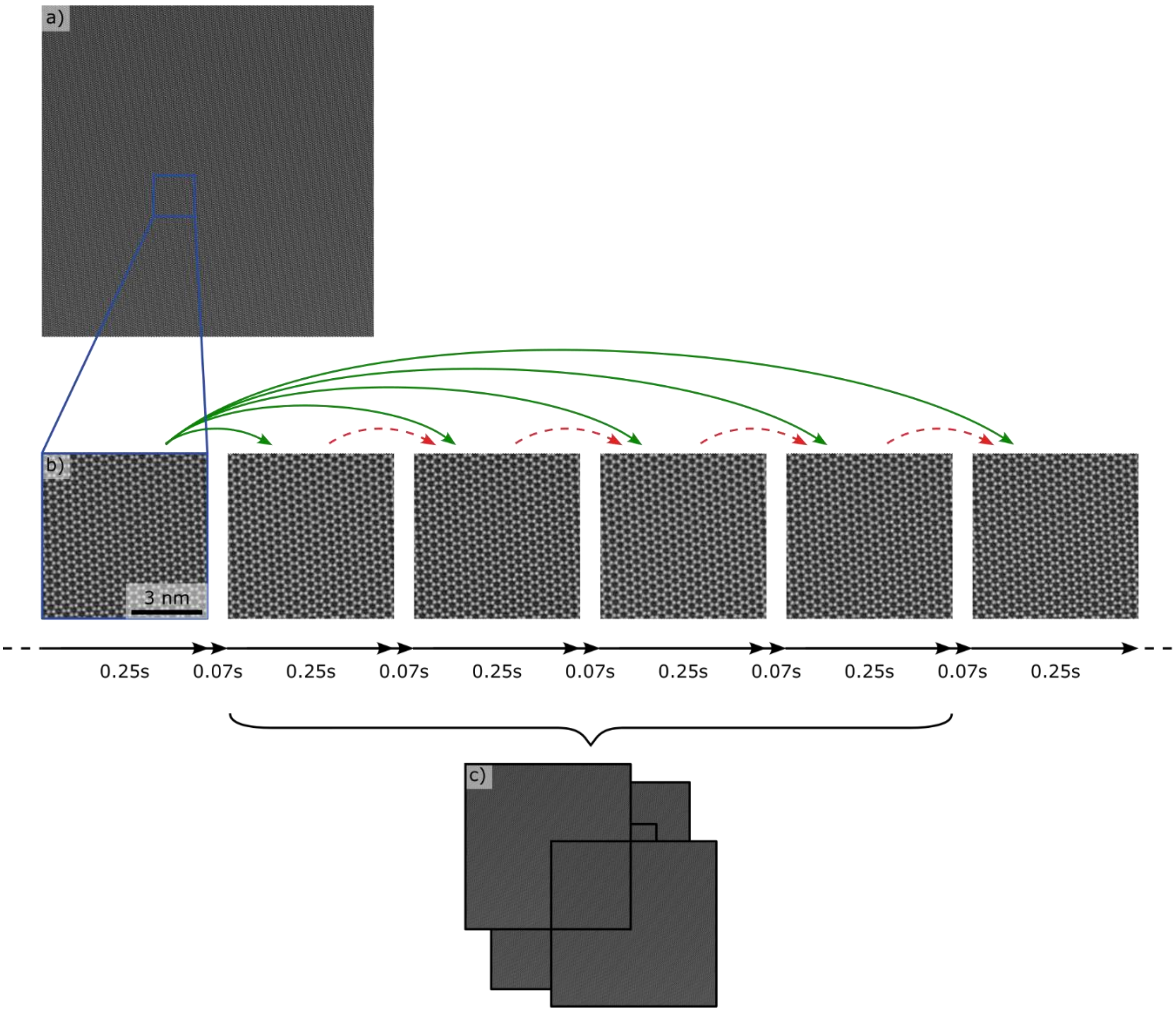

Figure S1: During the experiment, a video is recorded to enable tracking of the total dose on the investigated area. To get a live view while searching for a clean area for investigation and focusing at reasonable speed, an integration time of 0.25s per frame was selected. To increase the signal-to-noise ratio in the actual image series, four to eight frames are added after a movie alignment procedure. This is described here: (a) A full field of view image taken from the movie recorded during the measurement, with the region of interest (ROI) highlighted in blue. (b) In a first alignment step, consecutive frames are aligned with each other (red dashed arrows). In the second alignment step, all ROI images are aligned to one reference frame (green solid arrows). The overall shift finally is applied to the full field of view images. The sample was

continuously irradiated during the experiment. (c) Four (monolayer), six (bilayer) or eight (trilayer) frames are added to each other. The shifts are exaggerated for illustration reasons, in reality, they are smaller than the lattice constant.

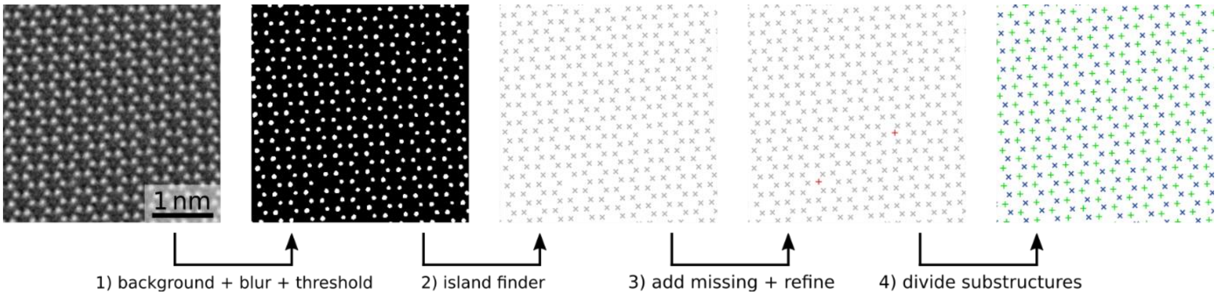

Figure S2: The identification of atomic column positions is demonstrated using an exemplary area within an image of monolayer MoS_2, though the procedure can be applied to bi- and trilayer analogously. (1) A threshold is applied to separate the atomic column positions into distinct islands. Beforehand, to ensure uniformity across the entire field of view, the raw image is first normalized by dividing it by its local intensity to remove background intensity gradients. Additionally, a Gaussian blur is employed to reduce noise in the raw image using. (2) An island finder algorithm is used to generate a list of approximate positions for most atomic columns. (3) The detection of atomic column positions, which may be missing due to their low intensities, is conducted; added atomic column positions are marked in red. (4) The two substructures are finally separated, indicated by blue and green markers, respectively. For further details and visualizations of the detection of missing atomic column positions and the separation into substructures, refer to Figure S3.

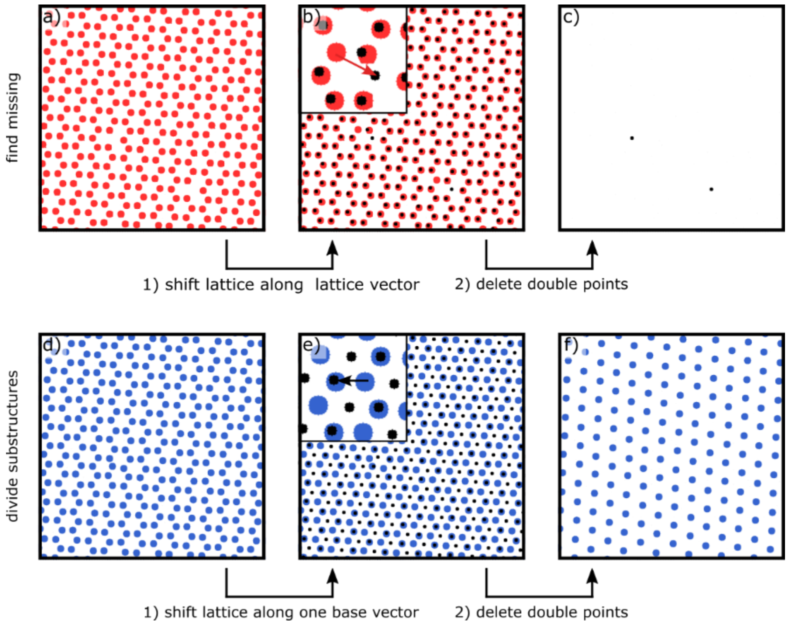

Figure S3: Top row: Procedure to identify missing atomic column positions. (a) Incomplete lattice with missing atomic column positions. (b) To identify missing atomic column positions, the lattice is shifted by the lattice vector (red arrow in inset). (c) Atomic column positions of the shifted lattice (black), which are close to original atomic column positions (red), are deleted. The remaining atomic column positions in the shifted lattice are those, missing in (a). Bottom row: Procedure to separate sub-structures: (d) Complete lattice. (e) The lattice is shifted by the base vector (black arrow in inset). (f) Atomic column positions of the original lattice (blue), which are close to atomic column positions of the shifted lattice (black), are deleted. The remaining atomic column positions (blue) refer to the first substructure. All atomic column positions of the original lattice that are not included in the first substructure, constitute the second substructure.

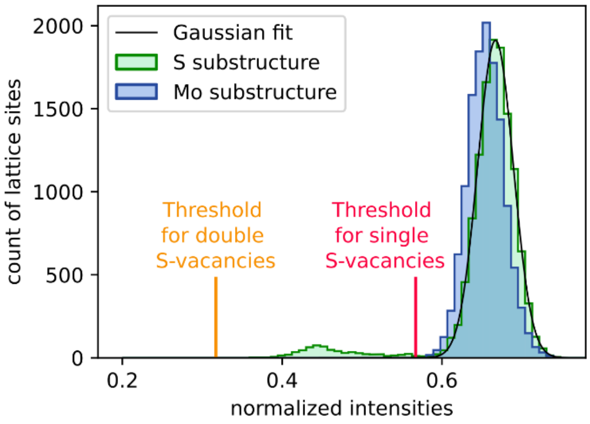

Figure S4: Calculation of the intensity thresholds from Figure 2 in the main text by Gaussian fitting. The Gaussian fit of intensities within the S substructure is shown in black. The thresholds are established below the mean value of this Gaussian fit at a fixed intensity difference. The intensity difference may require adaption for different HRTEM image series.

Supplementary Information to Figure 3

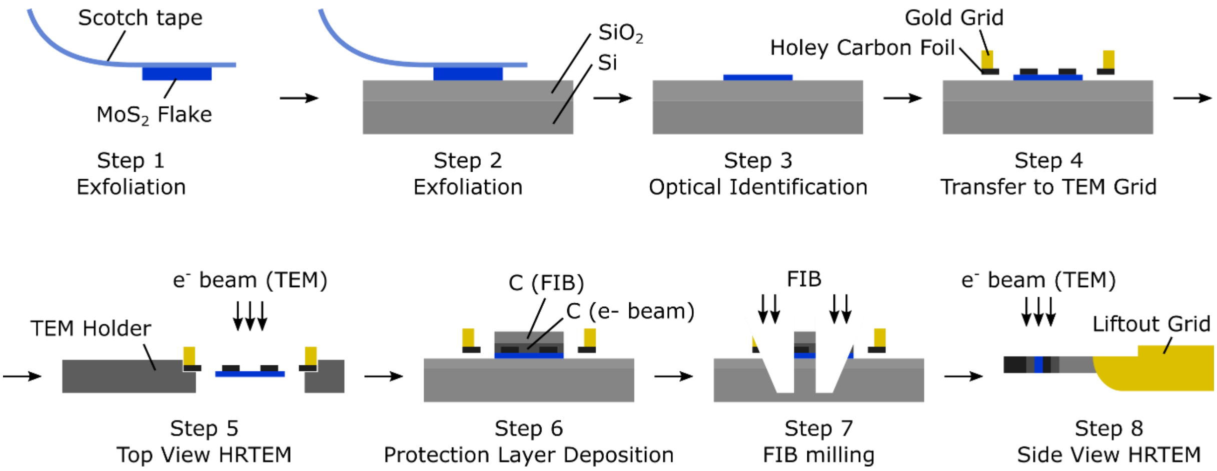

Figure S5: Step-by-step sample preparation to record plan and cross-sectional view HRTEM images from the same 1-3-layer MoS_2 flake. Step 1: The Scotch tape method is used do thin down MoS_2 flakes through exfoliation. Step 2: To deposit thin parts of the flakes, the tape is brought into contact with a Si substrate, which is coated with a 90nm layer of SiO_2.Step 3: A suitable flake is identified based on optical contrast using a light microscope. Step 4: A gold TEM grid with a holey carbon film is placed over the MoS_2 flake and the flake is transferred to the grid as described in the methods section of the main text. Step 5: The TEM-grid with the attached flake is inserted into the TEM with a special TEM holder and plan view HRTEM image series are recorded on mono-, bi- and tri-layer regions. Step 6: The grid is placed on a new silicon wafer that features a natural 3 nm SiO_2 layer. By drop casting and evaporation of a droplet of isopropyl alcohol, the grid is pulled into close contact with the substrate. The flake region is identified using SEM and then protected by depositing a layer of amorphous carbon via a gas injection system (GIS). Initially, a thin layer is slowly deposited using the electron beam, followed by a thicker layer deposited using the FIB. Step 7: A cross-section of the flake is cut out using the FIB, lifted out by a micromanipulator, and attached to a lift-out grid. It is then further thinned down by the FIB. Step 8: The cross-sectional view HRTEM images can now be recorded.

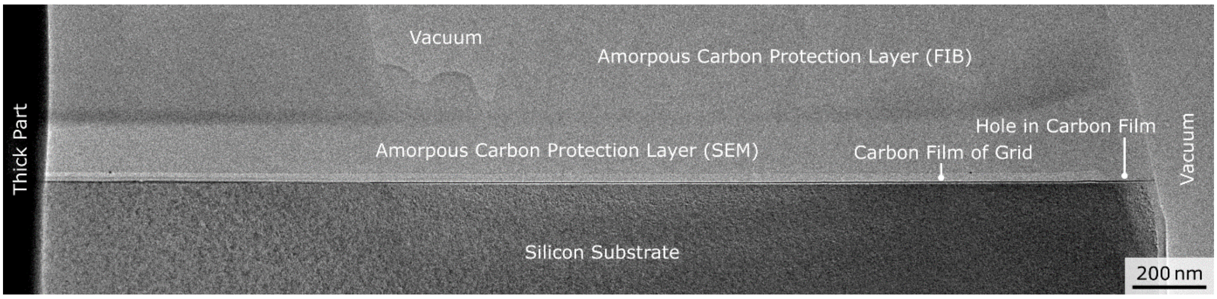

Figure S6: Detailed labels to Figure 4 of the main text. The three amorphous carbon layers are marked. On the right, an edge of a hole in the carbon film of the grid can be seen.

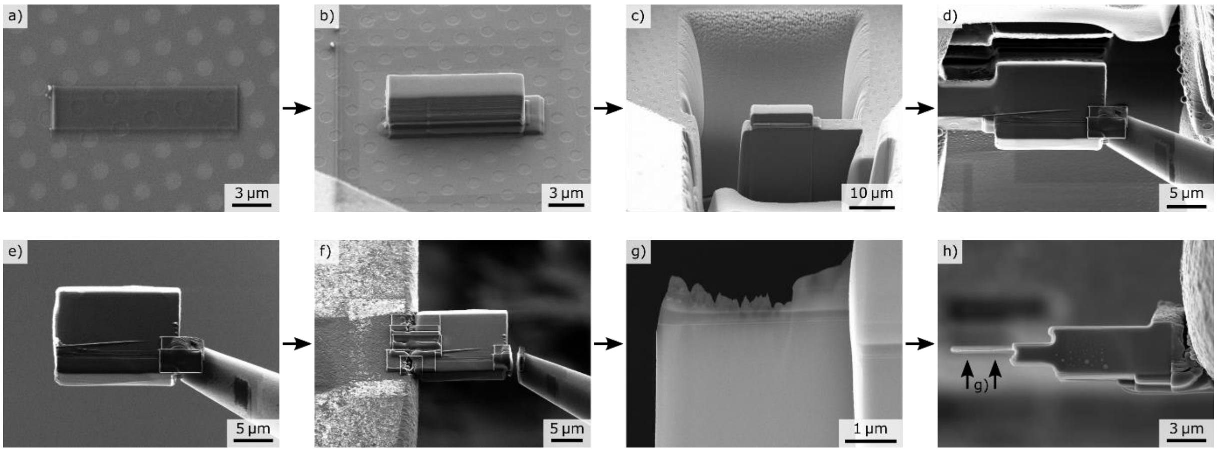

Figure S7: Secondary electron images of the FIB lamella sample preparation steps in the SEM/FIB Zeiss NVision 40 Ar. (a) A thin carbon layer, deposited on the sample area of interest with the electron beam and a gas injection system (GIS). (b) A thick amorphous carbon layer deposited by the FIB and a GIS. (c) The vertical cross-section (lamella) cut from three sides by the FIB. (d) The micromanipulator connected to the lamella by small carbon sheets, deposited by the FIB. (e) The lamella fully cut from all sides and lifted out from the initial sample (f) The lamella connected to the TEM grid and cut off from the micromanipulator. (g) Side view of the thinned FIB lamella and (h) plan view.

Supplementary Information to Figure 4

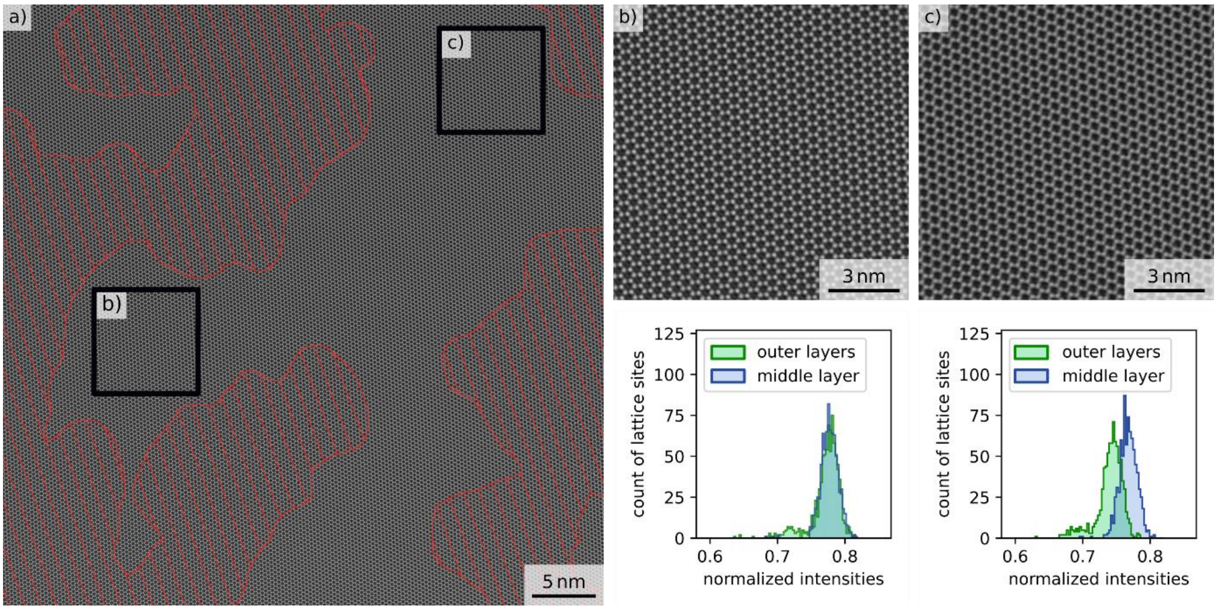

Figure S8. The effect of small variations in defocus due to slight sample bending making local normalization of the extracted intensities necessary. (a) 80kV C_C/C_S-corrected HRTEM image of trilayer MoS_2 with clear variation of imaging conditions over the whole field of view. Contaminated areas are crosshatched in red and excluded from the analysis. (b) Exemplary area with optimal defocus and the corresponding atom intensity histogram calculated only from this area. (c) Exemplary area slightly off from optimal defocus and the respective histogram. Comparing both histograms in (b) and (c), there is a noticeable shift between them. These shifts can be corrected by locally normalizing the intensity at each lattice point relative to the intensities of surrounding lattice points. With this local normalization the broadening of the peaks in the intensity histogram calculated from the whole field of view can be reduced. The variation of imaging conditions shown here is more pronounced than in the images evaluated in Figure 5 of the main text.

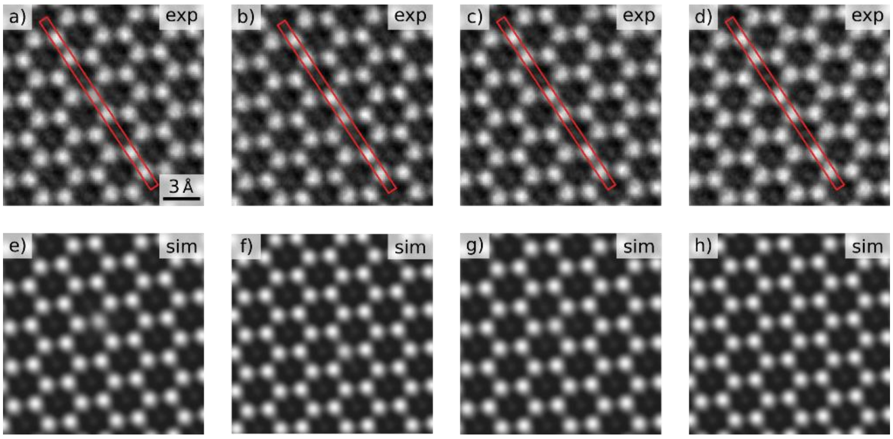

Figure S9: Experimental (upper row) and simulated (lower row) Cc/Cs-corrected 80kV HRTEM images of trilayer MoS_2 with different defects. The region used to generate the line plots in Figure 4 of the main text is highlighted with a red frame. The defects are hardly visible, as the change in intensity caused by one missing atom in a column of several atoms is very small. The aberrations obtained from the experimental PCTF, used for the simulation are $C_3 = -7\,\mu m$, $A_1 = 1.05\,nm$, $A_2 = 15$ nm and $B_2 = 10\,nm$.

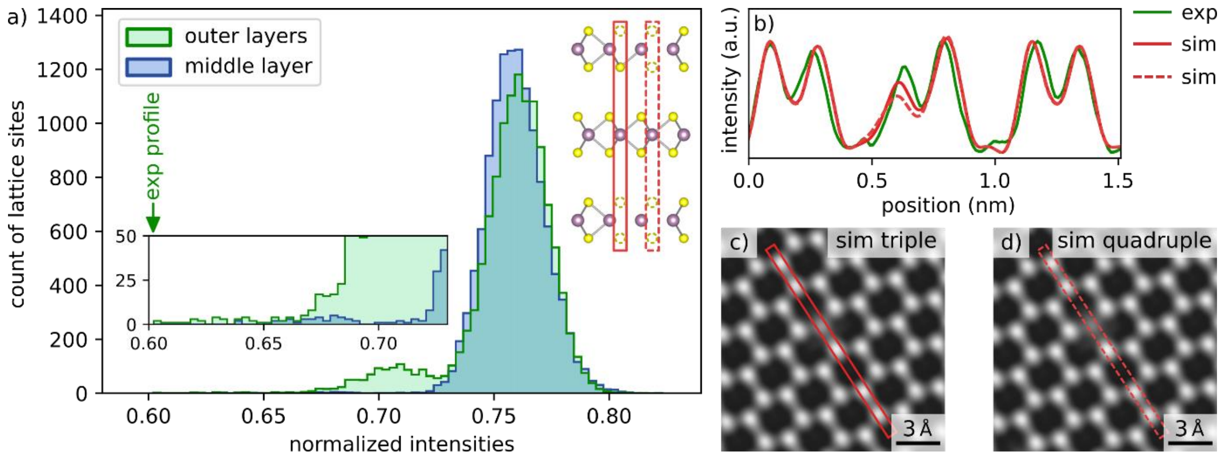

Figure S10: Comparison of the experiment with simulations to rule out the presence of triple and quadruple S vacancies in trilayer MoS_2. (a) Atom column intensity histogram from Figure 4 in the main text with the lowest present atomic column intensity marked by a vertical arrow. The atomic structure model shows the hypothetical situation of a triple S vacancy (red solid frame) and a quadruple S vacancy (red dashed frame). (b) Intensity profiles over the lowest experimentally observed intensity (green line), the simulated triple S vacancy (red solid line) and quadruple S vacancy (red dashed line). The intensities of triple and quadruple S vacancies are considerably lower than the lowest observed intensity, ruling out the presence of triple and quadruple S vacancies in our experimental data. (c) HRTEM image simulations[7] of triple and quadruple (d) S vacancies in trilayer MoS_2. The region of the profiles is marked in red.

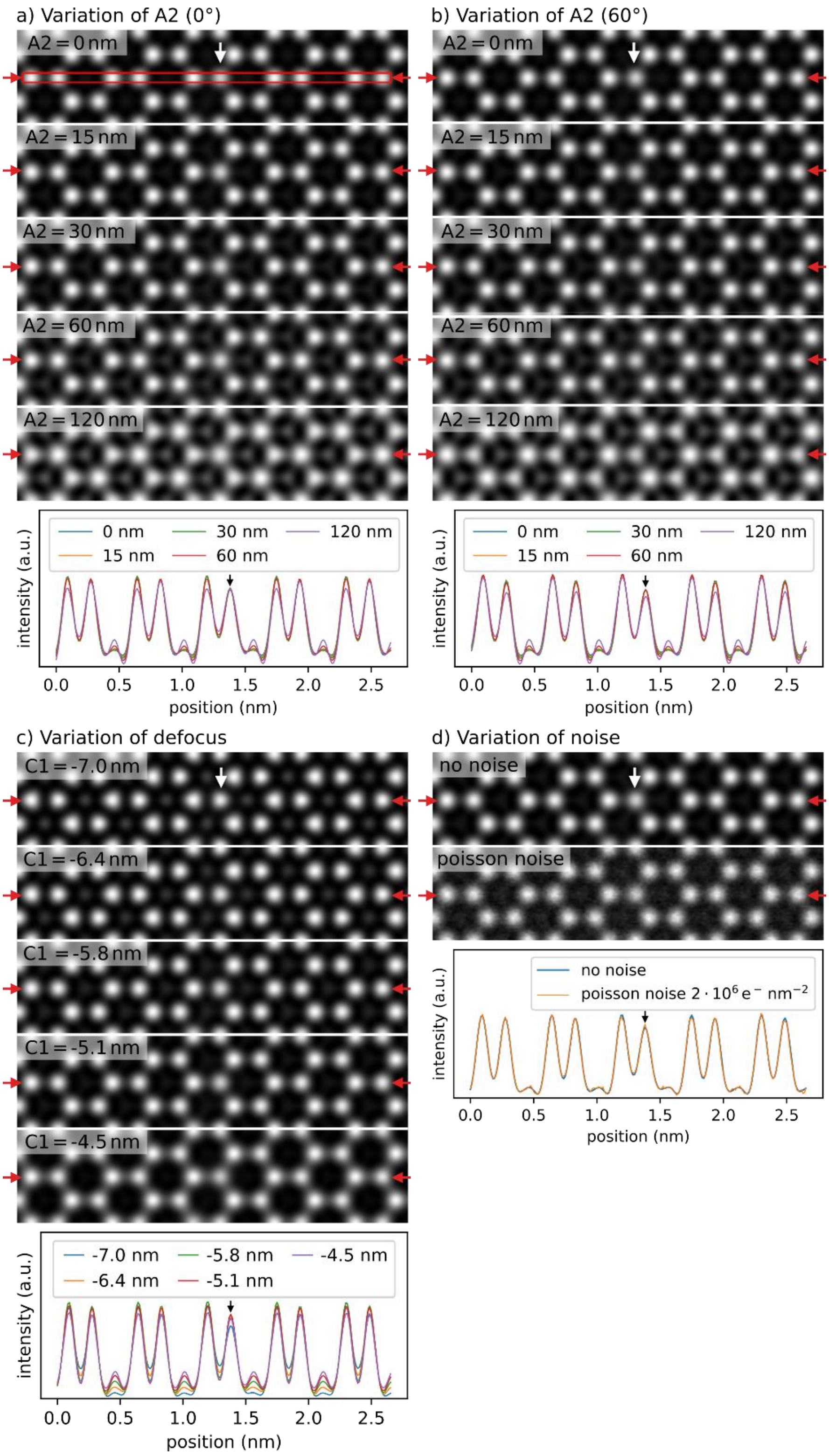

Figure S11: HRTEM image simulations[7] of trilayer MoS_2 featuring a single sulfur vacancy in the middle layer (indicated by a white/black arrow). Unless otherwise specified, the parameters are C1 = -6.3 nm, C3 = 7 µm, with all other aberrations set to zero and no noise added. Even with reasonable variations in aberrations or noise, the line plots clearly differentiate between intact atomic columns and a sulfur vacancy. (a), (b) Variation of threefold astigmatism. Specific combinations of the magnitude and orientation of the threefold astigmatism allow for independent variation of the intensities of both substructures, which can even be reversed. An absolute value of A2 higher than 30 nm can be ruled out based on our experiments. (c) Variation of defocus within an experimentally reasonable range. (d) The influence of noise, simulated at a dose rate of $2 \times 10^6\ e^- nm^{-2} s^{-1}$ is very low and does not lead to misinterpretations of the intensities. For all simulations, the pixel size is consistent with that used in the experiments in this work. The red box exemplarily highlights the area of the first line plot. Red arrows mark line plots in all images. White/black arrows mark the horizontal position of the vacancy.

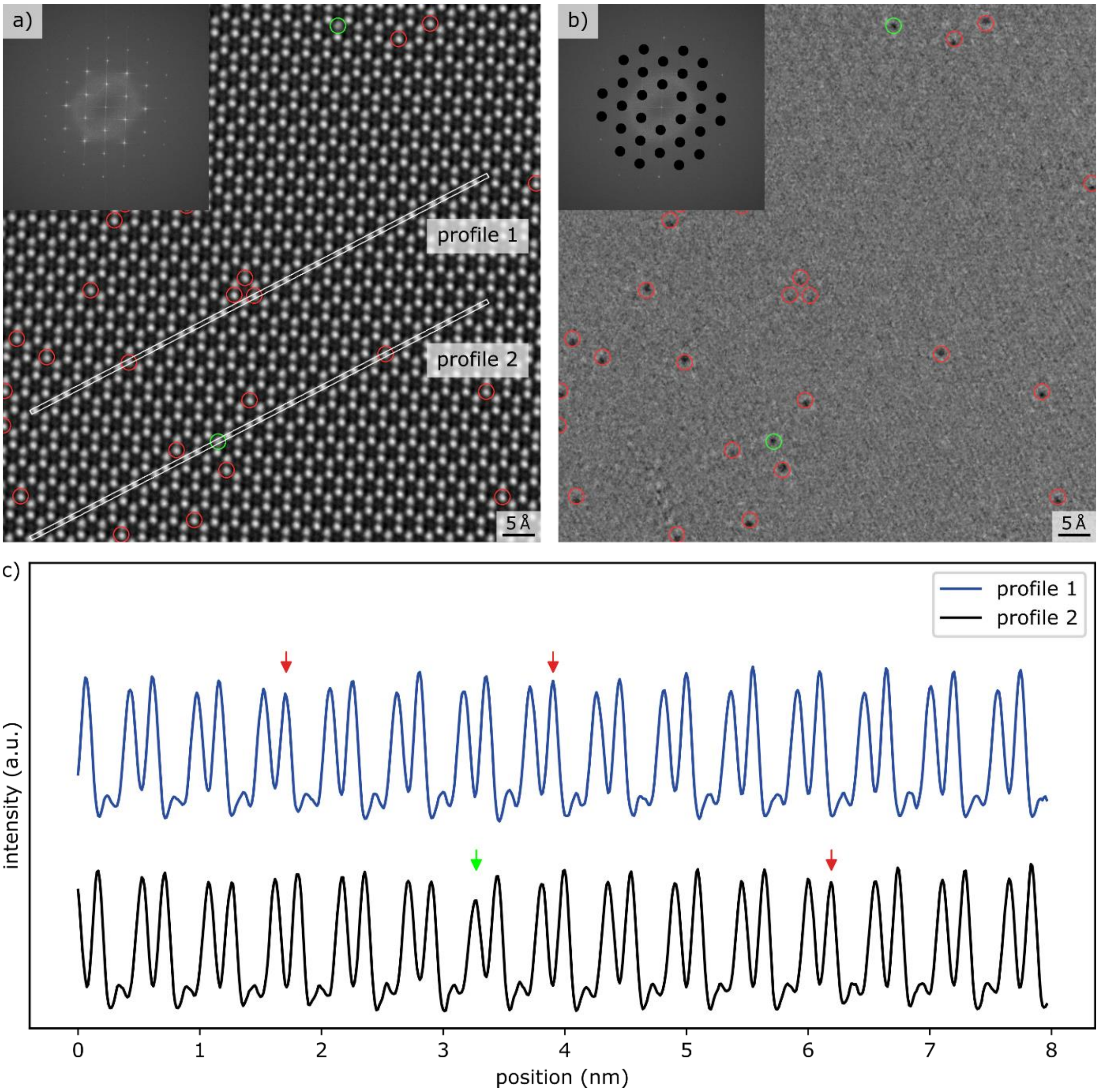

Figure S12: (a) Cutout of an experimental 80kV C_C/C_S-corrected HRTEM image of trilayer MoS_2 with marked defect positions, automatically calculated with the routine, presented in this work. The green and red circles mark sulfur vacancies in the outer layers and middle layer, respectively. In the inset the central region of the FFT is shown. (b) By removing the Bragg reflection spots from the FFT, a Fourier-filtered version of (a) is created. Here small changes in the lattice periodicity can be seen as dark spots. The positions of the dark spots are in good agreement with the detected defects from the intensity histogram method. (c) Line scans along the white frames in (a). The defect positions are marked with arrows in the respective color.

Supplementary Information to Figure 5

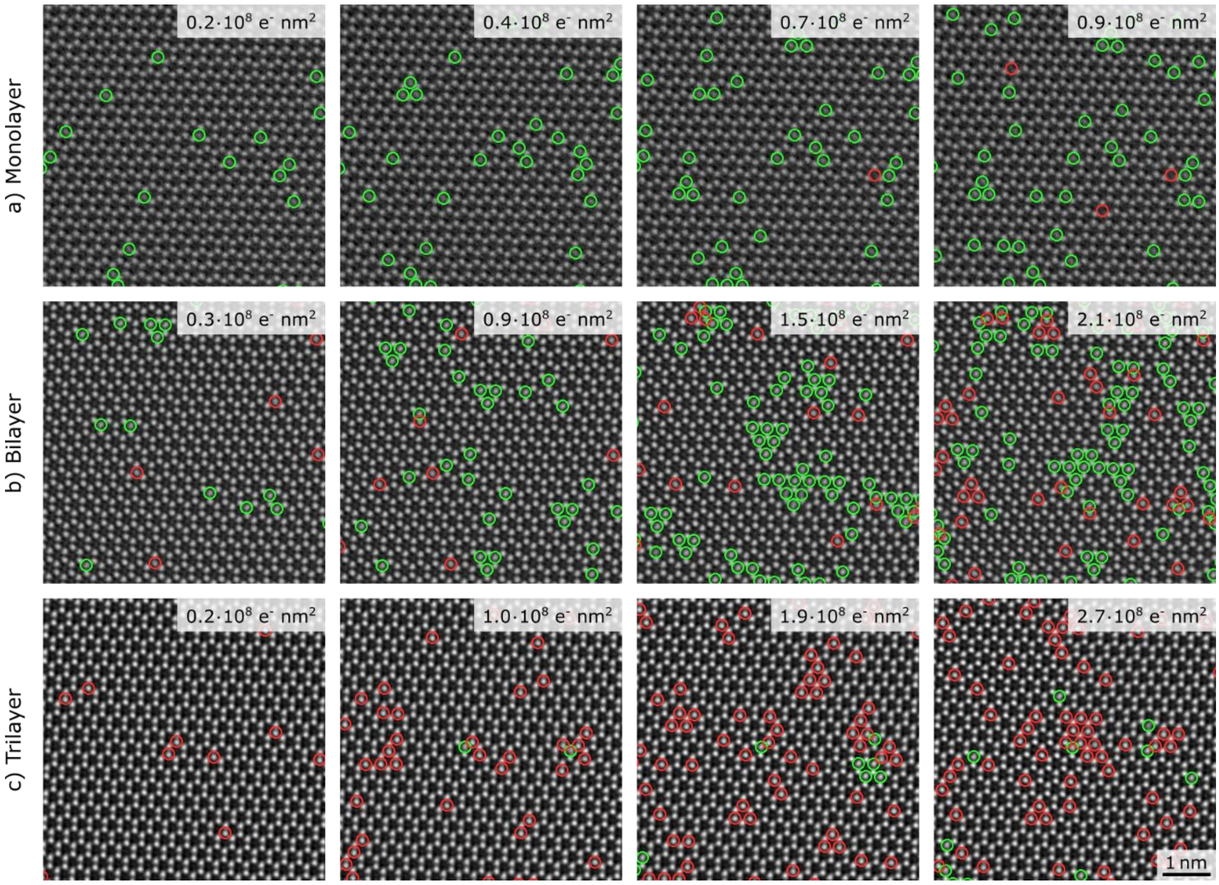

Figure S13: Exemplary 80kV C_C/C_S-corrected HRTEM images, cropped from the HRTEM image series of MoS_2 evaluated in Figure 5 of the main text. The green and red circles mark sulfur single and double vacancies in the case of a monolayer (a), sulfur vacancies in the bottom and the top layer in the case of a bilayer (b) and sulfur vacancies in the middle layers and outer layer in the case of a trilayer (c), respectively. The exposure before acquiring the first frame is due to navigation to the target area and focusing. The images are obtained from an alignment procedure as described in Figure S1, combining four frames for the monolayer, six frames for the bilayer and eight frames in the trilayer series. This is because the thicker the material, the slower defects are created and the finer are the intensity differences caused by defects.

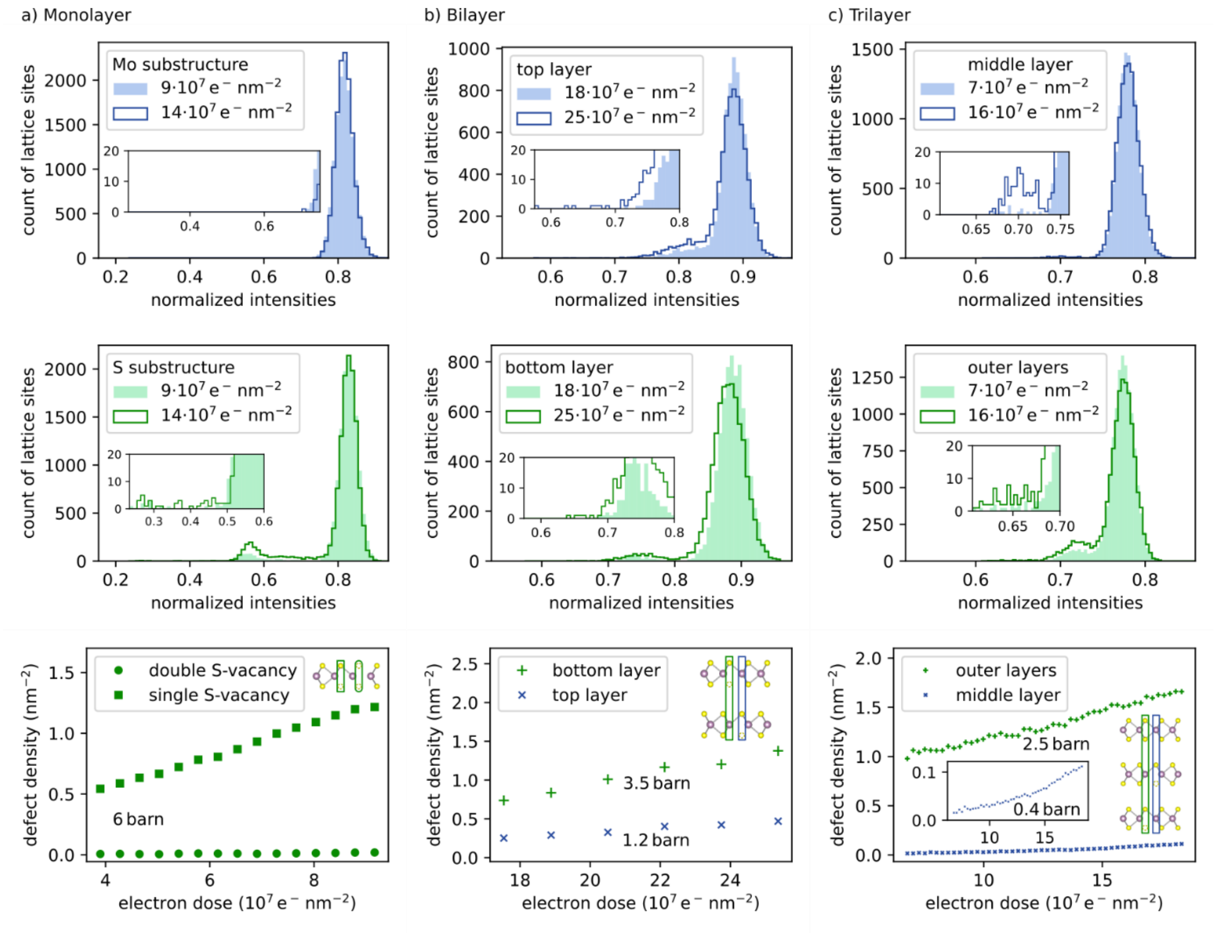

Figure S14: Reproduction of the results from Figure 5 of the main text on other MoS_2 flakes. Crystal structures designed with VESTA. [6]

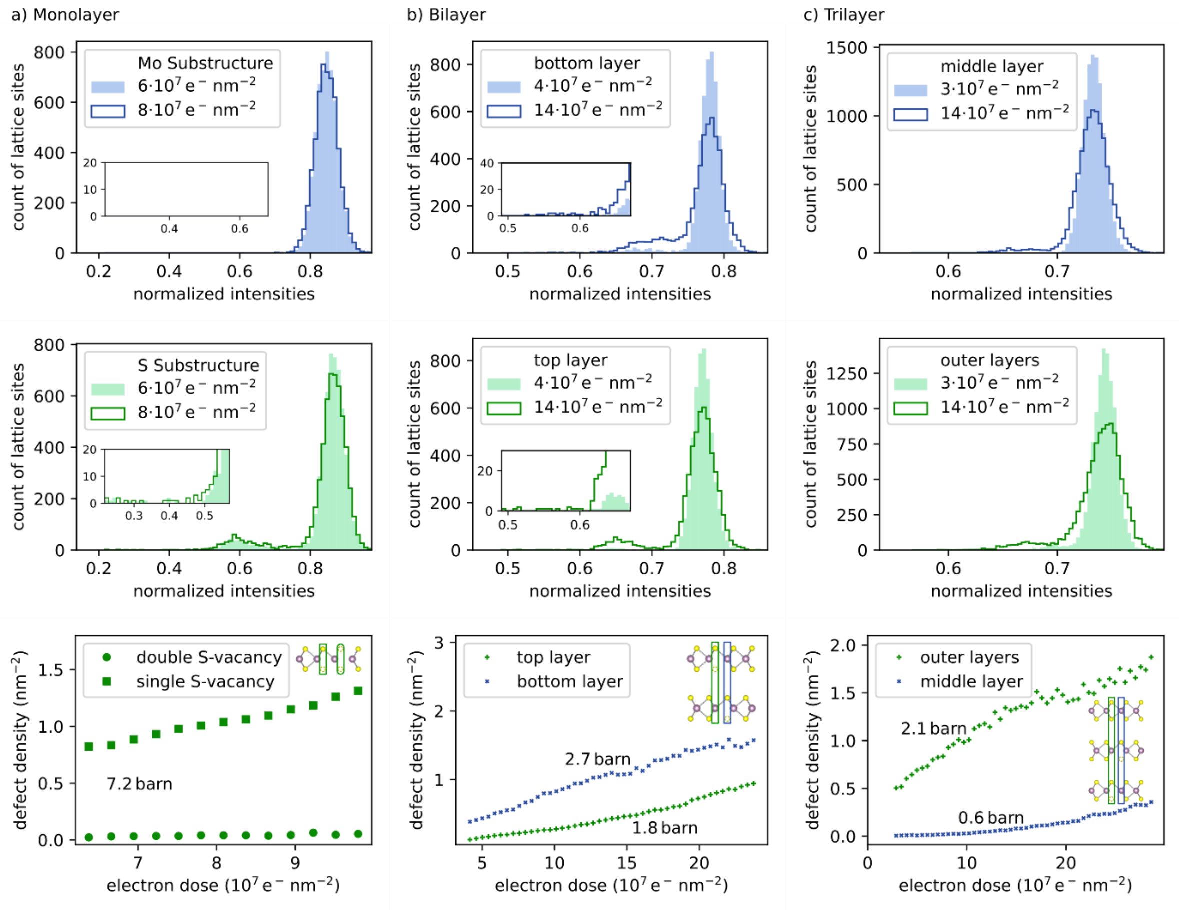

Figure S15: Reproduction of the results from Figure 5 of the main text with other HRTEM image series on the same MoS_2 flake. Crystal structures designed with VESTA.[6]

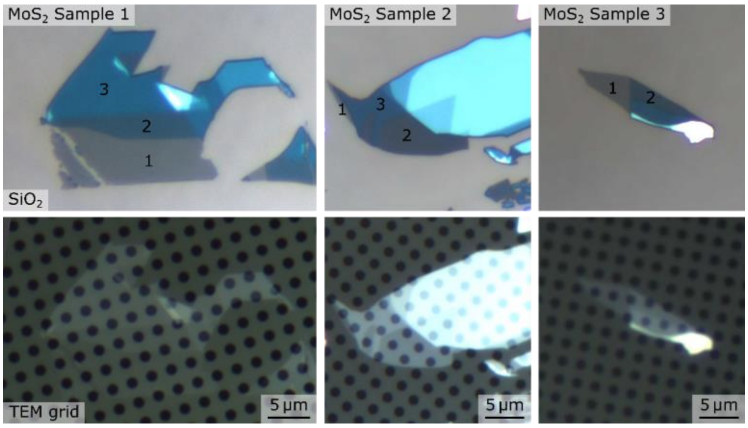

Figure S16: Light optical images of the samples used in this work, as exfoliated on a 90nm SiO_2/Si substrate and prepared for TEM experiments on the grid. The layer number obtained by optical contrast is denoted by the numbers in the figure. The data for Figures 3, 5 of the main text and Figures S6, S7, S12, S13 and S15 was recorded on sample 1. The data for Figure 4 of the main text and Figures S8, S9 and the trilayer series in S14 was recorded on sample 2. The data for Figure 2 of the main text and Figures S1-3 and the mono- and bilayer series in S14 were recorded on sample 3.

Literature

[1] Hai Li, Jumiati Wu, Xiao Huang, Gang Lu, Jian Yang, Xin Lu, Qihua Xiong, and Hua Zhang: Rapid and Reliable Thickness Identification of Two-Dimensional Nanosheets Using Optical Microscopy ACS Nano 2013, 7, 11, 10344–10353
https://doi.org/10.1021/nn4047474

[2] M M Benameur, B Radisavljevic, J S Héron, S Sahoo, H Berger and A Kis: Visibility of dichalcogenide nanolayers 2011 Nanotechnology 22 125706
https://doi.org/10.1088/0957-4484/22/12/125706

[3] Jannik C. Meyer, C. O. Girit, M. F. Crommie, A. Zettl; Hydrocarbon lithography on graphene membranes. Appl. Phys. Lett. 24 March 2008; 92 (12): 123110.
https://doi.org/10.1063/1.2901147

[4] Linck, M.; Hartel, P.; Uhlemann, S.; Kahl, F.; Müller, H.; Zach, J.; Haider, M.; Niestadt, M.; Bischoff, M.; Biskupek, J.; Lee, Z.; Lehnert, T.; Börrnert, F.; Rose, H.; Kaiser, U. Chromatic Aberration Correction for Atomic Resolution TEM Imaging from 20 to 80 kV. Phys. Rev. Lett. 2016, 117, 076101.
https://doi.org/10.1103/PhysRevLett.117.076101

[5] Tibor Lehnert, Mahdi Ghorbani-Asl, Janis Köster, Zhongbo Lee, Arkady V. Krasheninnikov, and Ute Kaiser: Electron-Beam-Driven Structure Evolution of Single-Layer MoTe2 for Quantum Devices, ACS Appl. Nano Mater. 2019, 2, 5, 3262–3270 https:
//doi.org/10.1021/acsanm.9b00616

[6] Momma, Koichi, and Fujio Izumi. "VESTA 3 for three-dimensional visualization of crystal, volumetric and morphology data." Journal of applied crystallography 44.6 (2011): 1272-1276
https://doi.org/10.1107/S0021889811038970

[7] Madsen J and Susi T. The abTEM code: transmission electron microscopy from first principles [version 2; peer review: 2 approved]. Open Res Europe 2021, 1:24
https://doi.org/10.12688/openreseurope.13015.2
